# P02.41. Yoga for musculoskeletal conditions: a systematic review of intervention protocols

**DOI:** 10.1186/1472-6882-12-S1-P97

**Published:** 2012-06-12

**Authors:** L Ward, S Stebbings, D Cherkin, D Baxter

**Affiliations:** 1 School of Physiotherapy, University of Otago, Dunedin, New Zealand; 2 School of Medicine, University of Otago, Dunedin, New Zealand; 3 Group Health Research Institute, Seattle, USA

## Purpose

To review intervention protocols of yoga for musculoskeletal conditions.

## Methods

Twenty-one databases were electronically searched using database-specific search strings incorporating “yoga”, “musculoskeletal”, “back pain”, “arthritis” and “random”. Inclusion criteria were full-text articles of randomised controlled trials, involving yoga as a primary intervention for clinically-diagnosed musculoskeletal conditions in adults. Articles were assessed for methodological quality and risk-of-bias.

## Results

Fifteen articles are included in the systematic review. Articles represent five musculoskeletal conditions, and vary from pilot studies to trials evaluating efficacy and effectiveness of yoga for musculoskeletal conditions. Quality ratings range from 1-8 on the PEDro scale, and 4-17 on the van Tulder scale. Pilot studies tend to show an unclear or high risk of bias, whereas trial studies are predominantly low risk. Heterogeneity in content and reporting of intervention protocols is seen across and within musculoskeletal conditions, as follows. Six styles of yoga were represented, with some protocols based on one style and others an amalgam of different styles. Thirteen interventions were in an outpatient setting (duration 6-24 weeks), and two were in a residential setting (duration 1 week). Hours spent in yoga sessions ranged from 1-56 hours per week and 8-72 hours per intervention. The majority of trials incorporated yoga posture, breathing and relaxation techniques. However, the posture content between trials was difficult to compare at face value due to variations in naming of a posture across different styles of yoga.

## Conclusion

Heterogeneity in the content and reporting of yoga intervention protocols makes comparison of results difficult across studies. To address this challenge of heterogeneity, future research should address which components of a yoga intervention protocol can be standardised, and define a range of variation within which an intervention may still be considered sufficiently homogenous to enable comparison across different research trials.

